# Cost-effective clinical trial design: Application of a Bayesian sequential model to the ProFHER pragmatic trial

**DOI:** 10.1177/17407745211032909

**Published:** 2021-08-18

**Authors:** Martin Forster, Stephen Brealey, Stephen Chick, Ada Keding, Belen Corbacho, Andres Alban, Paolo Pertile, Amar Rangan

**Affiliations:** 1 Department of Statistical Sciences ‘Paolo Fortunati’, University of Bologna, Bologna, Italy; 2 Department of Economics and Related Studies, University of York, York, UK; 3 York Trials Unit, Department of Health Sciences, University of York, York, UK; 4 Technology & Operations Management Area, INSEAD, Fontainebleau, France; 5 Department of Economics, University of Verona, Verona, Italy; 6 Department of Health Sciences, University of York, York, UK; 7 Faculty of Medical Sciences & NDORMS, University of Oxford, Oxford, UK; 8 James Cook University Hospital, Middlesbrough, UK

**Keywords:** Bayesian decision-theoretic model, sequential clinical trial, cost-effectiveness analysis

## Abstract

**Background/Aims:**

There is growing interest in the use of adaptive designs to improve the efficiency of clinical trials. We apply a Bayesian decision-theoretic model of a sequential experiment using cost and outcome data from the ProFHER pragmatic trial. We assess the model’s potential for delivering value-based research.

**Methods:**

Using parameter values estimated from the ProFHER pragmatic trial, including the costs of carrying out the trial, we establish when the trial could have stopped, had the model’s value-based stopping rule been used. We use a bootstrap analysis and simulation study to assess a range of operating characteristics, which we compare with a fixed sample size design which does not allow for early stopping.

**Results:**

We estimate that application of the model could have stopped the ProFHER trial early, reducing the sample size by about 14%, saving about 5% of the research budget and resulting in a technology recommendation which was the same as that of the trial. The bootstrap analysis suggests that the expected sample size would have been 38% lower, saving around 13% of the research budget, with a probability of 0.92 of making the same technology recommendation decision. It also shows a large degree of variability in the trial’s sample size.

**Conclusions:**

Benefits to trial cost stewardship may be achieved by monitoring trial data as they accumulate and using a stopping rule which balances the benefit of obtaining more information through continued recruitment with the cost of obtaining that information. We present recommendations for further research investigating the application of value-based sequential designs.

## Introduction and background

There is growing interest in the use of adaptive designs to improve the efficiency of clinical trials. Adaptive designs involve monitoring outcome data as they accumulate, permitting changes to be made to the trial – such as varying the allocation ratio, or stopping early – in response to the evolving evidence. A large literature surveys their development and application and the potential they offer for improving efficiency.^1–8^

Despite this interest, little attention has been paid to how statistical decision rules in an adaptive clinical trial might formally account for the costs and benefits of the trial itself. This hampers assessment of the value that such designs might create for health care systems. A growing number of theoretical papers, some with illustrative applications, have proposed the use of value-based criteria for fixed sample size designs,^9–11^ as well as adaptive ones.^12,13^ However, lack of guidance on how research costs should be measured and how accumulating evidence about treatment costs and health outcomes may inform decision rules as a trial progresses, means that incorporation of costs and benefits in adaptive clinical trials remains an under-researched area. The United Kingdom’s National Institute for Health Research has recognised this and has recently provided ‘Annual Efficient Studies’ funding to clinical trials units to investigate further. The ‘Costing Adaptive Trials’ (CAT) project^14^ will provide costing guidance; the ‘EcoNomics of Adaptive Clinical Trials’ (ENACT) project^15^ will assess how cost-benefit criteria may be incorporated.

In this article, we apply a recent contribution proposing a Bayesian decision-theoretic model of a sequential clinical trial^13,16^– (a sequential trial is a special kind of adaptive trial in which data are monitored as they accumulate over a sequence of interim analyses – using retrospective data from the) PROximal Fracture of the Humerus: Evaluation by Randomisation (ProFHER) pragmatic trial. The ProFHER trial was a multicentre randomised clinical trial conducted in the United Kingdom National Health Service which compared surgery with sling immobilisation for the treatment of displaced proximal humeral fracture.^17–19^ We believe that the application is the first of its kind to use research cost data to inform this model. It is presented as a ‘proof of concept’ study which contributes to the gap in the literature discussed above.

The ProFHER trial was designed according to standard criteria for a fixed sample size clinical trial. However, by considering how the effectiveness and research cost data accumulated over the course of the trial, we can estimate when the trial could have stopped, had a decision rule based on evaluating the cost-effectiveness of the research process been used. Our interest is not in whether such a rule could replace a fixed sample size, or group sequential, clinical trial designed according to traditional criteria. Rather, we are interested in whether such a rule could complement such designs, by providing additional information to trials teams about whether interim evidence suggests that the benefit of randomising further patients into the trial is worth the cost. This matter is of particular interest for trials such as the ProFHER trial, where the extra costs associated with surgery and subsequent revision and secondary surgery, compared with the cheaper alternative of sling immobilisation, meant that while accumulating clinical evidence may not have suggested that one treatment was superior to the other, accumulating cost-effectiveness evidence might have done. Further, patient and surgeon preferences for the two sharply contrasting treatment options were expected to be a major threat to completing successfully recruitment into the trial. Hence a value-based stopping rule might have been useful.

## Methods

### The Bayesian model

Chick et al.^13^ model a two-armed sequential clinical trial in which patients are randomised, in a pairwise and sequential manner, to a new health technology, N, and a control (or standard) health technology, S. Follow-up of health outcomes and treatment costs for each patient occurs after Δ≥0 units of time. To reflect beliefs concerning the cost-effectiveness of the technologies before starting the trial, the model places a prior distribution on the expected value of the net monetary benefit of N minus that of S, where net monetary benefit for technology i∈{N,S} is defined as $\lambda E_{i}-C_{i}$ , where *E* is a random variable denoting effectiveness, *C* is a random variable denoting treatment cost and *λ* is the willingness to pay for an additional unit of effectiveness in the jurisdiction of interest (e.g. following advice for the United Kingdom National Health Service,^20^ the ProFHER trial set *λ* equal to £20,000 per Quality Adjusted Life Year (QALY)).

The objective of the model is to obtain a rule to halt recruitment to the trial. This rule maximises the expected net benefit of carrying out the trial and then recommending one of the two technologies on cost-effectiveness grounds for the treatment of *P* patients who are expected to benefit from the adoption decision. The costs of carrying out the trial and the costs incurred in switching technologies are included in the measure of expected net benefit. The Supplemental Material discusses the model’s objective function in more detail.

The trial can make a maximum number of $Q_{\max\;\;}$ pairwise allocations. The outcome of interest is incremental net monetary benefit, *X*, the difference between the net monetary benefit of N and S. For pairwise allocation *j*, $j=1,2,\ldots ,Q_{\max\;\;}$ , this is

(1) $$X_{j}=\lambda \left(E_{N,j}-E_{S,j}\right)-\left(C_{N,j}-C_{S,j}\right)$$

We assume that *X* has a normal distribution and that its expected value, *W*, is unknown and its variance, $\sigma_{X}^{2}$ , is known. Before starting the trial, beliefs about *W* are modelled using a normal prior distribution with an expected value of $\mu_{0}$ and variance of $\sigma_{0}^{2}$ . $n_{0}=\sigma_{X}^{2}/\sigma_{0}^{2}$ is the ‘effective sample size’, measured in pairwise allocations, of the prior distribution.

Assuming a fixed rate of recruitment to the trial, we may express the delay in terms of time, Δ, or pairwise allocations, τ≥0 . The trial comprises three distinct stages:

1. Stage I: patients are recruited and randomised, but no patient-level health outcome or treatment cost data are observed owing to the delay in following up;
2. Stage II: patient-level health outcome and treatment cost data are observed and are used to update the prior distribution using Bayes’ rule. There is the option to randomise another pair of patients, or to stop recruitment to the trial. Define *x* as an observation of incremental net monetary benefit. Then the posterior mean for expected incremental net monetary benefit after outcomes for *n* pairwise allocations is^21^

$$\mu_{n}=\frac{\mu_{0}n_{0}+\sum_{j=1}^{n}x_{j}}{n_{0}+n}$$

If, during Stage II, the expected benefit of randomising a further pair of patients is less than the cost, Stage II finishes, having made *T* pairwise allocations, and the trial moves to Stage III.

1. Stage III: health outcome and treatment cost data for patients in the ‘pipeline’– those who have been treated but whose outcomes are yet to be observed – are observed and Bayesian updating continues.

*T* is chosen so that the overall expected value of the trial – the total incremental expected benefit which accrues to the *P* patients, minus the fixed $\left(c_{\mathrm{fixed}}\right)$ and variable (*c*) research costs, together with any costs *I* incurred in adopting one of the two technologies – is maximised. The decision rule fully accounts for the uncertainty in the data generating process and the prior distribution for expected incremental net monetary benefit. We call a rule which meets this objective an ‘Optimal Bayes Sequential policy’ and obtain such a policy using dynamic programming methods.^13^

There are two scenarios in which it is not optimal to enter Stage II: (1) the expected benefit from entering Stage II is less than that of running a trial with a fixed number of pairwise allocations in the range (0,τ) . In this scenario, the Optimal Bayes Sequential policy selects the same sample size as a trial designed to maximise the difference between the expected value of sample information and the cost of sampling.^9–11^ We call this an ‘Optimal Bayes One Stage’ design; (2) the value of the prior mean favours one of the two technologies so strongly that the expected cost of conducting any trial outweighs the expected benefit. In this scenario, the Optimal Bayes Sequential policy is to run no trial and base the adoption decision on the sign of the prior mean alone.

**Figure 1.**
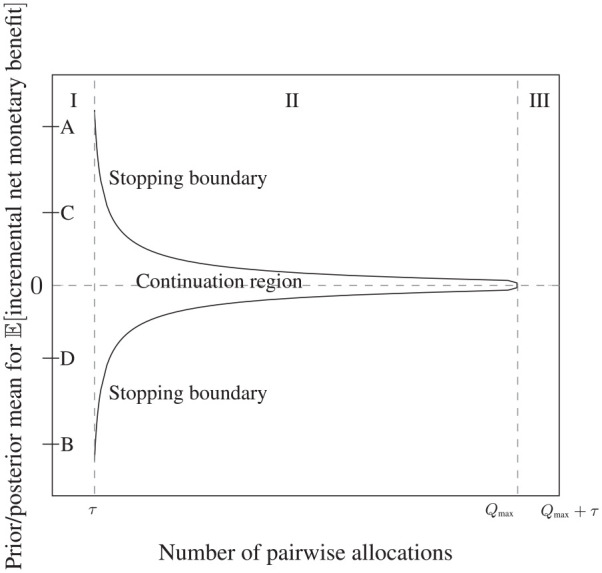
Stopping boundary for the Optimal Bayes Sequential model, showing the three stages of the trial (marked ‘I’, ‘II’ and ‘III’) and the continuation region. Stages II and III are shown assuming that the sequential trial stops at the maximum sample size of $Q_{\max}$ pairwise allocations. *τ* is the delay, measured in terms of the number of pairwise allocations, in observing the health outcome and treatment cost for each pairwise allocation. Interim analyses to inform early stopping are permitted during Stage II as outcomes are observed.

Figure 1 presents a representation of the stopping policy for the problem in (pairwise allocations × prior/posterior mean) space. If it is optimal to run a sequential trial, recruitment of patients takes place during Stage I but no outcomes are observed. At the start of Stage II, health outcomes and treatment costs for the first pairwise allocation are observed and used to update the prior mean. Outcomes then arrive sequentially, the posterior mean is updated sequentially and interim analyses of the data are permitted. As long as the posterior mean lies within the area defined by the stopping boundary (we refer to this as the ‘continuation region’), it is optimal to continue recruitment. Once the posterior mean crosses the boundary, it is optimal to halt recruitment and move to Stage III. There is no longer a continuation region in Stage III because recruitment is no longer taking place. In the analysis that follows, for consistency with the ProFHER application, we assume that the cost of switching technologies, *I*, is equal to zero. This means that, once outcomes for all patients in the trial have been observed, the cost-effectiveness of the new technology is judged according to whether or not the posterior mean is greater than zero. If it is greater than zero, the new technology is deemed to be cost-effective; if not, the standard technology is deemed to be cost-effective.

The letters ‘A’ to ‘D’ in Figure 1 denote ranges for the prior mean $\mu_{0}$ which define the optimal choice of trial design. If $\mu_{0}$ lies between points ‘C’ and ‘D’, it is optimal to run the sequential trial, with the starting point for the path of the posterior mean in Stage II being determined by the value of $\mu_{0}$ . If $\mu_{0}$ lies between ‘A’ and ‘C’ or ‘D’ and ‘B’, it is optimal to run the Optimal Bayes One Stage design. If $\mu_{0}$ lies above A or below B, no trial should be run and the adoption decision should be based on the value of the prior mean alone: above A, prior information is strong enough to favour immediate adoption of N; below B, it is strong enough to favour immediate adoption of S.

The shape of the stopping boundary and the ranges for $\mu_{0}$ over which each of the three trial designs is optimal are a function of the model’s parameter values and so will vary across applications. Where there exists a large degree of uncertainty over the values of a particular parameter, sensitivity analysis may be carried out.^13^

### The application

#### The ProFHER trial

Between September 2008 and April 2011, 250 patients aged 16 years and older who presented to orthopaedic departments in United Kingdom National Health Service hospitals with a displaced proximal humeral fracture were randomised to either (1) surgical treatment, which consisted of fracture fixation with plate and screws to preserve the humeral head, or humeral head replacement, followed by active rehabilitation, or (2) non-surgical treatment, which consisted of sling immobilisation for the injured arm for as long as was thought necessary, followed by active rehabilitation. Following discussions with the funder, it was agreed that a single follow-up time point would not be specified for the primary health outcome measure, the Oxford Shoulder Score. Rather, follow-up points were fixed at 6, 12 and 24 months. Analysis of clinical and cost-effectiveness used the intention to treat principle (during the trial, 16 patients randomised to surgery switched to sling and 2 randomised to sling switched to surgery).^17–19^ The economic evaluation consisted of a cost-utility analysis which took the National Health Service perspective. The European Quality of Life-5 Dimensions-3L instrument was used to obtain the QALYs at 3, 6, 12 and 24 months using the area under the curve method.

The trial’s results suggested that there was no difference between surgical intervention and sling, as measured by the average value of the Oxford Shoulder Score at the three follow-up points. Surgical intervention for one patient cost an estimated £1758 more than sling (95% confidence interval = (£1126, £2389)) and yielded an estimated 0.0101 fewer QALYs (95% confidence interval = (−0.13, 0.11)). A 5-year follow-up found the main results unchanged.^22^

**Figure 2.**
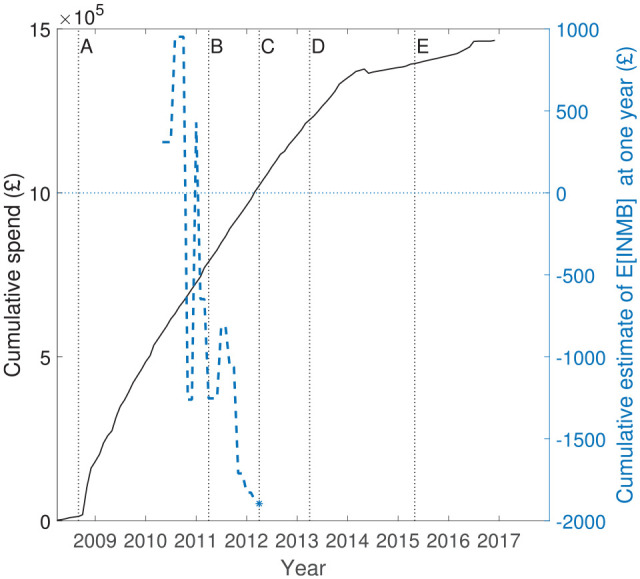
Cumulative budget spend for the ProFHER trial (left axis, continuous line) and average of incremental net monetary benefit at 1 year (right axis, dashed blue line, plotted in blocks of 10 patient pairs, 10 receiving surgery and 10 receiving sling). Key milestones: ‘A’– recruitment starts; ‘B’– recruitment finishes; ‘C’– 1 year follow-up finishes; ‘D’– 2 year follow-up finishes; ‘E’– publication of principal articles.^17,18^

The ProFHER trial was funded by the National Institute for Health Research, with a total budget of £1,485,585. Figure 2 shows how the research budget spend accumulated over the lifetime of the project (left axis, continuous black line), together with the path for the cumulative estimate of incremental net monetary benefit at 1 year (right axis, dashed blue line), measured in blocks of 10 patient pairs at a time.^*^ Positive values suggest that surgery is cost-effective. Key milestones in the project are denoted by the letters ‘A’ to ‘E’. The research costs plotted in Figure 2 are those relating to the research budget itself. Treatment costs were not charged to this budget, rather they were funded as part of normal commissioning arrangements within the National Health Service. For the purposes of this work, we assume that treatment costs would have been the same with or without the trial, on average, across the hospitals participating in the trial.

The path of the cumulative estimate of expected incremental net monetary benefit shown in Figure 2 was not available to the investigators as the trial progressed. The path shows that, although surgery appeared cost-effective initially, the estimate favoured sling by late 2010 and remained that way for the rest of the follow-up. Viewed in terms of incremental effectiveness versus incremental cost at 1 year, the overall story of the trial is that there was no evidence that surgery was more effective than sling (using both the primary health outcome measure and QALYs), but there was strong evidence that surgery was more costly than sling. The Supplemental Material provides further details about how the differences between the estimates of incremental QALYs, Oxford Shoulder Score and treatment costs evolved.

#### Estimation of parameter values

Using the research cost data from the trial, we estimated that costs of approximately £161,000 were incurred prior to the recruitment of the first patients in September 2008 (labelled as ‘A’ in Figure 2). During the recruitment phase (which finished in April 2011, labelled ‘B’) and the 2-year follow-up phase (which finished in April 2013, labelled ‘D’), further costs of approximately £1,020,000 were incurred. The main results^17,18^ were reported 2 years later (‘E’), and the project concluded at the end of December 2016. Approximately £289,000 of costs were incurred post follow-up. These covered the tasks of data preparation, cleaning, analysis and report writing. The total spend was approximately £1,470,000. We assume that the costs incurred during the recruitment and follow-up phases were split 50:50 between fixed and variable costs, which implies an estimate of an average cost per pairwise allocation of c=£4,080 .

For the purposes of exposition, we assume that the delay Δ is equal to 1 year. We estimate that the rate of recruitment is approximately 47 pairwise allocations per year, so that τ=47 pairwise allocations. We assume a near non-informative prior, setting $\mu_{0}=0$ and $n_{0}$ equal to two pairwise allocations, representing the lack of evidence for cost-effectiveness at the start of the trial. The other parameter values used for the application, together with their sources and the assumptions used to obtain them, are reported in Supplemental Table 2 of the Supplemental Material and accompanying discussion.

#### Implementation of the model

We take the perspective of the ProFHER researchers prior to commencing the trial, but post trial commissioning. That is, we assume that a decision to commission the research and commit fixed costs $c_{\mathrm{fixed}}$ has already been taken. The solution to the model permits interim analyses to be made at any point during Stage II, including one pairwise allocation at a time. For the purposes of illustration, we assume that interim analyses take place once every 10 pairwise allocations.

We run two versions of the model. The first assumes that the maximum number of pairwise allocations that can be made, $Q_{\max\;\;}$ , is equal to 125, that is, the sample size of the ProFHER trial itself. The second assumes that $Q_{\max\;\;}$ is equal to 250, that is, double this maximum sample size. We ran the latter version of the model to test the sensitivity of results to a design which permits the stopping time to exceed that of the ProFHER trial. Matlab code which implements the computations is provided at https://github.com/sechick/htadelay.

## Results

### When would the Bayesian sequential version of the ProFHER trial have stopped?

**Figure 3.**
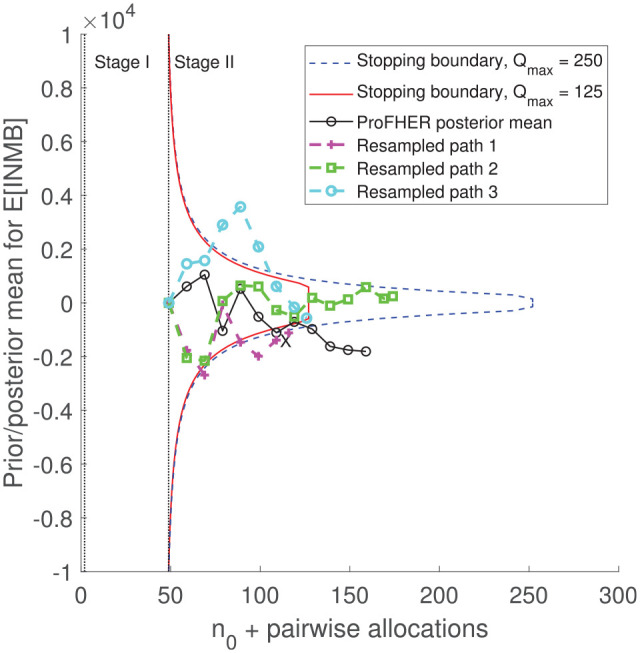
Stopping boundaries for the two versions of the model, together with the path for the posterior mean generated using the trial’s data (black line, marker: ‘°’) and three resampled paths from the bootstrap analysis (dashed lines, markers: ‘+’, ‘□’ and ‘°’). X marks the first interim analysis at which the posterior mean lies outside the stopping boundary (for both versions of the model).

Figure 3 plots the Stage II stopping boundaries for the two versions of the model. Also drawn is the path of the posterior mean for expected incremental net monetary benefit, derived using the data as it accumulated in the ProFHER trial (continuous black line, markers: ‘°’). This is drawn using the summary data for effectiveness and treatment costs from the trial, arranged in blocks of 10 pairwise allocations, and reported in Supplemental Table 1 of the Supplemental Material. The other paths in Figure 3 are described in the next section. Figure 3 shows that doubling the maximum sample size from 125 to 250 pairwise allocations has little impact on the shape and location of the stopping boundary between the start of Stage II and $Q_{\max\;\;}=125$ .

The first point on the path for the posterior mean, at the start of Stage II and at an effective sample size of 49 pairwise allocations (equal to $n_{0}=2$ plus the delay of 47 pairwise allocations), is equal to the prior mean $\left(\mu_{0}=0\right)$ . Figure 3 shows that, independently of whether $Q_{\max\;\;}=125$ or 250, Stage II would have concluded after 107 patient pairs had been recruited, with a posterior mean equal to −£1110. This is shown by the interim analysis marked ‘X’ in Figure 3 and corresponds to the first point at which the posterior mean lies outside the stopping boundary. Follow-up of the 47 patient pairs in the pipeline is shown by the remaining circles on the path and would have led to a posterior mean for expected incremental net monetary benefit equal to approximately −£1810, suggesting that surgery is not cost-effective. Hence, irrespective of whether $Q_{\max\;\;}$ is set to be 125 or 250 pairwise allocations, the sequential trial would have stopped early, with no change in the technology recommendation and little change in the estimate of cost-effectiveness, saving 18 patient pairs (14% of the trial’s actual sample size) and approximately 18×£4,080=£73,000 (5% of the total cost of the trial).

### Bootstrap analysis

To investigate the degree of variability in the sample size and other operating characteristics, we used a non-parametric bootstrap analysis. We sampled at random, and with replacement, from the data in Supplemental Table 1 of the Supplemental Material and obtained 5000 bootstrapped paths for the posterior mean. For each path, we compared the posterior mean with the stopping boundary, assuming it would be practical to run interim analyses in blocks of 10 pairwise allocations. For each interim analysis, we established when Stage II would have stopped, as well as the adoption decision, cost of the trial and the posterior mean for expected incremental net monetary benefit at the end of Stage III. Three bootstrapped paths are shown in Figure 3 for a trial with $Q_{\max\;\;}=125$ , with interim and follow-up analyses marked. Resampled Path 3 (cyan and marked ‘°’) stops the trial at the third interim analysis, having crossed the upper part of the boundary; resampled Path 1 (magenta and marked ‘+’) stops at the second interim analysis, having crossed the lower part of the boundary; and resampled Path 2 (green and marked ‘□’) runs to the maximum sample size. Paths 1 and 3 suggest that sling is cost-effective at the end of follow-up (the posterior mean is negative); Path 2 suggests that surgery is cost-effective (the posterior mean is positive).

Some operating characteristics are summarised in Table 1, labelled ‘bootstrap’. They show that, when $Q_{\max\;\;}=250$ , the average sample size of the Optimal Bayes Sequential design is 77 pairwise allocations (minimum 57; maximum 250), 38% lower than the trial’s actual sample size. The expected saving in the trial’s budget resulting from the reduced sample size is estimated to be £196,000 (13% of the research budget). The posterior mean for expected incremental net monetary benefit at the end of Stage III is estimated to be −£1853. This design recommended sling for 92% of the bootstrapped paths, with 82% of the paths stopping having first crossed the lower part of the stopping boundary. Also shown in Table 1 are the operating characteristics for a fixed sample size trial in which each resampled path in the bootstrap analysis runs to $Q_{\max\;\;}=250$ pairwise allocations: 99.3% of paths conclude with a recommendation of sling, but this improvement is achieved at a cost of approximately £706,000 ((250 − 77) × £4080) when compared with the Optimal Bayes Sequential design.

**Table 1.** Results for the 5000 resampled paths from the bootstrap and Monte Carlo analysis.

	Average	% change	Standard deviation	Minimum	Maximum	
$Q_{\max\;\;}=250$						
*Optimal Bayes Sequential*						
Sample size (pairwise allocations) – bootstrap	77	−38	27	57	250	
Sample size (pairwise allocations) – Monte Carlo	88	−30	20	57	250	
Change in budget (£000) – bootstrap	−196	−13	110	−277	510	
Change in budget (£000) – Monte Carlo	−151	−10	82	−277	510	
Posterior mean for cost-effectiveness(£)– bootstrap	−1853	–	1322	−5900	3046	
Posterior mean for cost-effectiveness(£)– Monte Carlo	−1820	–	449	−4190	−617	
*Fixed sample size*						
Sample size (pairwise allocations)	250	–	0	250	250	
Posterior mean for cost-effectiveness	−1832	–	720	−4047	1017	
$Q_{\max\;\;}=125$						
*Optimal Bayes Sequential*						
Sample size (pairwise allocations) – bootstrap	73	−42	19	57	125	
Sample size (pairwise allocations) – Monte Carlo	84	−33	16	57	125	
Change in budget (£000) – bootstrap	−210	−14	78	−277	0	
Change in budget (£000) – Monte Carlo	−167	−11	63	−277	0	
Posterior mean for cost-effectiveness(£)– bootstrap	−1845	–	1347	−5778	3670	
Posterior mean for cost-effectiveness(£)– Monte Carlo	−1811	–	460	−3900	−451	
*Fixed sample size*						
Sample size (pairwise allocations)	125	–	0	125	125	
Posterior mean for cost-effectiveness	−1804	–	988	−4951	2100	
	$Q_{\max\;\;}=250$			$Q_{\max\;\;}=125$		
Bootstrap	Sling	Surgery	Total	Sling	Surgery	Total
*Optimal Bayes Sequential*						
First crossing lower part of stopping boundary	0.815	0.020	0.835	0.805	0.023	0.828
First crossing upper part of stopping boundary	0.102	0.063	0.165	0.106	0.066	0.172
Total	0.917	0.083	1	0.911	0.089	1
*Fixed sample size*						
Total	0.993	0.007	1	0.961	0.039	1

Percentage changes in sample size reported in Column 3 are calculated as (a−125)/125 × 100, where *a* is the relevant average value from Column 2 and 125 refers to the number of pairwise allocations in the ProFHER trial. For rows which report a percentage change in the budget, the percentage refers to the change in the number of pairwise allocations, (a−125) , multiplied by the cost per pairwise allocation (£4080), expressed as a percentage of the total budget of £1,470,000.

Figure 3 showed that there is very little difference between the stopping boundary when the maximum sample size is reduced to that used in the ProFHER trial itself ( $Q_{\max\;\;}=125$ pairwise allocations). Table 1 shows that, when $Q_{\max\;\;}=125$ , the expected sample size falls by four pairwise allocations, from 77 to 73; the trial saves slightly more of the budget (£210,000) and the model shows sling to be cost-effective for 91% of the bootstrapped paths, which is little change from the 92% when $Q_{\max\;\;}=250$ .

**Figure 4.**
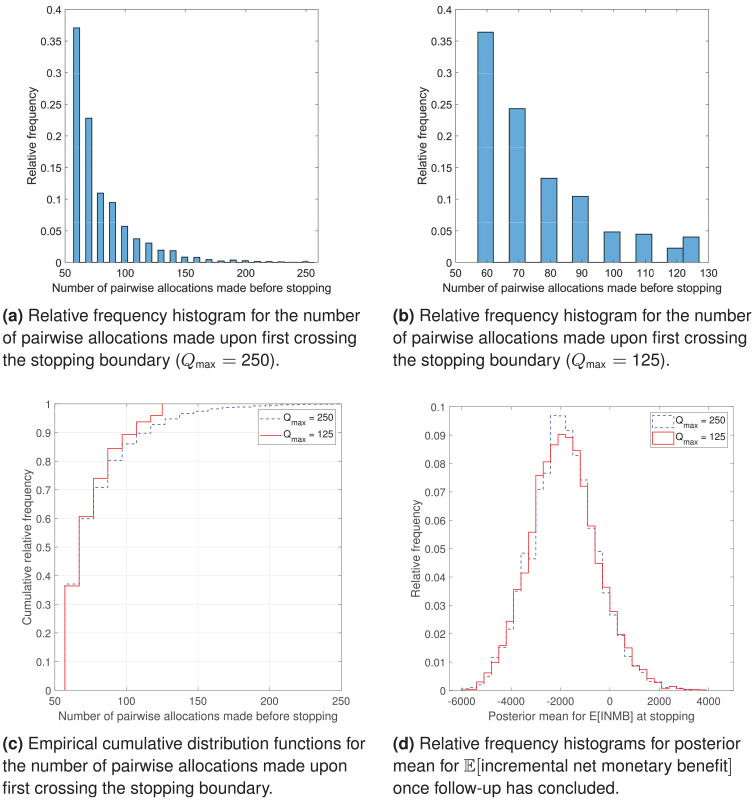
Graphical analysis of the bootstrap results: (a) relative frequency histogram for the number of pairwise allocations made upon first crossing the stopping boundary $\left(Q_{\max\;\;}=250\right)$ , (b) relative frequency histogram for the number of pairwise allocations made upon first crossing the stopping boundary $\left(Q_{\max\;\;}=125\right)$ , (c) empirical cumulative distribution functions for the number of pairwise allocations made upon first crossing the stopping boundary and (d) relative frequency histograms for posterior mean for E[incrementalnetmonetarybenefit] once follow-up has concluded.

Figure 4 presents some graphical summaries of the bootstrap analysis. Figure 4(a) shows that, when $Q_{\max\;\;}=250$ , approximately 37% of the resampled paths stop the trial at the first interim look and approximately 23% stop it at the second interim look, so that approximately 60% of bootstrapped paths have a sample size that is approximately half of the one used in the ProFHER trial (Figure 4(c)). Reducing $Q_{\max\;\;}$ to 125 pairwise allocations makes little difference (Figure 4(b) and (c)). Figure 4(d) shows that the relative frequency histograms for the posterior mean for expected incremental net monetary benefit at adoption are almost identical and appear slightly right-skewed.

### Sensitivity analysis

To investigate the sensitivity of our main results to different assumptions about how the data accumulated over the course of the trial, we also carried out a Monte Carlo simulation which took repeated draws of incremental net monetary benefit from a normal distribution with expected value equal to the value that was used for the bootstrap (approximately −£1808) and $n_{0}=2$ . Results are also presented in Table 1 and labelled ‘Monte Carlo’. Averages are qualitatively in line with those of the bootstrap analysis and standard deviations are smaller. For example, when the maximum sample size of the trial is set to 250 pairwise allocations, the expected sample size of the trial is 30% lower (compared with 38% lower in the bootstrap), the reduction in the budget is 10% (compared with 13%) and the posterior mean for expected incremental net monetary benefit is almost unchanged.

A discussion of further sensitivity analysis is presented in the Supplemental Material.

## Discussion and conclusion

With growing interest in the use of adaptive clinical trials, there is a need to explore how new approaches perform, from both economic and statistical perspectives. Our application of a Bayesian decision-theoretic model of a sequential clinical trial to the ProFHER pragmatic trial suggests that it could have stopped the trial early, saving about 5% of the research budget. The bootstrap analysis suggests that the sample size would have been reduced by approximately 38%, saving around 13% of the budget, with a probability of 0.92 of making a technology recommendation consistent with that of the trial itself. It also shows a large degree of variability in the trial’s sample size.

It is important to note that, although the model may be applicable in a range of trial settings with a pragmatic element, it will not be applicable to all trials. For example, it is unlikely to be suitable for Type C trials, which are more concerned with safety than with effectiveness or cost-effectiveness, as well as trials where the length of follow-up of the outcome of interest is close to the length of the recruitment period (these present little or no scope for using interim analyses) and trials where the health outcome measure of interest is the time to an event rather than a period of fixed duration (this breaks the model’s assumption about a fixed period of follow-up).

We conclude with some directions for future research:

1. Approximately 37% of resampled paths from the bootstrap analysis stop at the first interim analysis, with a sample size equal to just under half of that of the ProFHER trial. Given that about half of the surgeons who responded to a recent survey^23^ stated that they had changed practice because of ProFHER, it is unlikely that such a sample size will be deemed credible for changing practice. One extension would be to investigate the sensitivity of results to choice of follow-up period.
2. Some of the parameters used to populate the model are difficult to estimate, suggesting that additional sensitivity analysis is warranted. For example, the size of the population to benefit is a function of both the incidence rate and the time horizon over which an adoption decision applies. Defining fixed and variable costs may also be challenging, and the CAT project^14^ may provide helpful guidance. The costs of monitoring a sequential design may be higher than those for a fixed design.
3. The model assumes that patients are randomised in a pairwise manner to treatments, but there exists a large statistical literature on the use of allocation-adaptive randomisation in frequentist designs.^24^
4. The handling of missing data could be explored in further sensitivity analysis. This matter is being investigated as part of the ENACT project.^15^
5. The sampling variance is assumed to be known. This requires that either it be estimated at the start of the trial or that the methods of Chick et al.,^13^ Section 4, are used for the case of unknown sampling variance.
6. We assume a prior mean that is equal to zero and a prior variance which assigns a low weight to prior information. Choice of the prior mean is important because it determines whether no trial, a fixed sample size trial or a sequential trial are the preferred designs. It also affects the point at which the Stage II path for the posterior mean starts. Choice of the prior variance affects the weights placed on the prior information and the data. Although we believe that a non-informative prior is reasonable for the ProFHER trial, it may not be for other trials. This is another topic that is being investigated further in the ENACT project.^15^
7. The stopping boundary could be compared with a Bayesian design which uses a stopping rule based on the probability that a technology is cost-effective, together with frequentist group sequential stopping rules, as in Pertile et al.^12^

## Supplemental Material

sj-pdf-1-ctj-10.1177_17407745211032909 – Supplemental material for Cost-effective clinical trial design: Application of a Bayesian sequential model to the ProFHER pragmatic trial Click here for additional data file. Supplemental material, sj-pdf-1-ctj-10.1177_17407745211032909 for Cost-effective clinical trial design: Application of a Bayesian sequential model to the ProFHER pragmatic trial by Martin Forster, Stephen Brealey, Stephen Chick, Ada Keding, Belen Corbacho, Andres Alban, Paolo Pertile and Amar Rangan in Clinical Trials

## Notes

* The data are derived from Supplemental Table 1 of the Supplemental Material, ignoring the prior information and assuming that missing data are missing completely at random.^25^ The sequence of data used to allocate patients to blocks was arranged according to the date of the return. The sequence only had to be slightly amended to fit the pairwise comparisons approach. The mean of the absolute difference between the original rank in the sequence and the analysed rank in the pairwise sequence was 3.9 (SD: 2.2, median: 4, min: 0, max: 9).
