# Supplementary material for: Cost-effective clinical trial design: Application of a Bayesian sequential model to the ProFHER pragmatic trial
Source: Clin Trials. 2021 Aug 18;18(6):647–56. doi: 10.1177/17407745211032909 (PMC8592107; doi:10.1177/17407745211032909)
Supplement: sj-pdf-1-ctj-10.1177_17407745211032909 – Supplemental material for Cost-effective clinical trial design: Application of a Bayesian sequential model to the ProFHER pragmatic trial [file sj-pdf-1-ctj-10.1177_17407745211032909.pdf]

Not for publication

Cost-effective clinical trial design: application of a Bayesian sequential model to the ProFHER pragmatic trial. Supplemental material

Martin Forster[1], Stephen Brealey[2], Stephen Chick[3], Ada Keding[2], Belen Corbacho[2], Andres Alban[3], Paolo Pertile[4], and Amar Rangan[5]

[1]Department of Statistical Sciences “Paolo Fortunati”, Via Belle Arti 41, University of Bologna, Bologna, Italy and Department of Economics and Related Studies, University of York, Heslington, York YO10 5DD, United Kingdom

[2]York Trials Unit, Department of Health Sciences, University of York, York YO10 5DD, United Kingdom

[3]Technology and Operations Management Area, INSEAD, Fontainebleau, France 77300

[4]Department of Economics, University of Verona, Verona, Italy

[5]Department of Health Sciences, University of York, Faculty of Medical Sciences & NDORMS, University of Oxford and James Cook University Hospital, Middlesbrough, United Kingdom

18th June, 2021

1 Methods

1.1 The Bayesian model's objective function

The model of Chick et al. (2017a) assumed that no patients were being treated with technology N at the start of the trial and it was assumed that all patients would switch to it, were it found to be superior, in cost-effectiveness terms, to technology S. This assumption did not match the pragmatic nature of the ProFHER trial in that, prior to the start of the trial and owing to the absence of definitive clinical guidance, some patients in the population of interest were being treated with surgery and some with sling. Alban et al. (2020) propose an extension to account for mixed clinical practice: define p_{N} as the proportion of the P patients who would be treated with the new technology N (surgery, in the context of the ProFHER trial) in the absence of the trial. Hence $1 - p_{\mathrm{N}}$ is the proportion who would be treated with the standard S (sling), absent the

Table SM.1: Point estimates of QALYs and treatment cost data from the ProFHER trial, together with the number of observations used to obtain each point estimate, arranged in blocks of ten patient pairs. Also included is a row for the prior mean, together with the effective sample size of the prior (block = 0). In columns 6–9, block sizes with fewer than ten observations contain missing data.

	QALYs		Treatment costs		Number of observations			
Block	$\bar{E}_{\text{N}}$	$\bar{E}_{\text{S}}$	$\bar{C}_{\text{N}}$	$\bar{C}_{\text{S}}$	$n_{\bar{E}_{\text{N}}}$	$n_{\bar{E}_{\text{S}}}$	$n_{\bar{C}_{\text{N}}}$	$n_{\bar{C}_{\text{S}}}$
0 (Prior)	0	0	0	0	2	2	2	2
1	0.74	0.67	3166	2102	10	10	4	6
2	0.85	0.69	1855	47	10	9	6	6
3	0.66	0.84	2464	120	9	9	5	7
4	0.77	0.44	2191	1150	10	9	5	7
5	0.66	0.73	3921	32	10	10	4	5
6	0.68	0.78	2854	582	10	9	4	8
7	0.78	0.61	2549	1223	10	9	6	3
8	0.60	0.71	3081	3028	10	10	7	5
9	0.52	0.73	2689	20	10	10	5	5
10	0.75	0.81	2434	821	9	10	9	6
11	0.62	0.65	1918	27	10	9	5	3

trial. Define the adoption decision as $\mathcal{D} \in \{\text{S}, \text{N}\}$. A valid Stage II rule, or policy, π, takes the value of the posterior mean and the number of patient pairs already allocated and maps it to an action: whether to randomise another pair of patients or to stop the trial, follow up the pipeline patients, and make decision $\mathcal{D}$. The expected benefit from carrying out the trial is defined as:

$$\begin{aligned} V(\pi; \mu_0, n_0) = &- c_{\text{fixed}} + \\ &\mathbb{E}_\pi \left[-Tc + \mathbf{1}_{\mathcal{D}_{T+\tau}=\text{N}}(P(1-p_{\text{N}})W - I_{\text{N}}) + \mathbf{1}_{\mathcal{D}_{T+\tau}=\text{S}}(Pp_{\text{N}}(-W) - I_{\text{S}}) \,|\, \mu_0, n_0 \right], \end{aligned} \tag{SM.1}$$

where $\mathbf{1}_F = 1$ if F is true and zero otherwise and I_{N} and I_{S} are the costs of switching patients to N and S, respectively. $\mathbb{E}_\pi$ is the expectation induced by policy π and $T \in \{0, 1, \ldots, Q_{\text{max}}\}$ is the number of pairwise allocations made at the time of stopping. The fact that we assume that the switching costs are equal to zero and that the adoption decision must be one of either technology N or S means that the special case in section D.1.3 of Alban et al. (2020) applies and that the solution to Eq. (SM.1) simplifies to that of the problem considered by Chick et al. (2017a).

1.2 The application

1.2.1 Primary health outcome, quality of life and treatment cost analysis.

Table SM.1 presents the point estimates of mean QALYs and treatment costs, arranged in blocks of ten patient pairs, from the ProFHER trial. These data are used to plot the path for the point estimate of expected incremental net monetary benefit in Figure 2 of the journal article (ignoring block 0, which refers to the prior mean and its effective sample size), Figures SM.1a and SM.1b and for the bootstrap analysis.

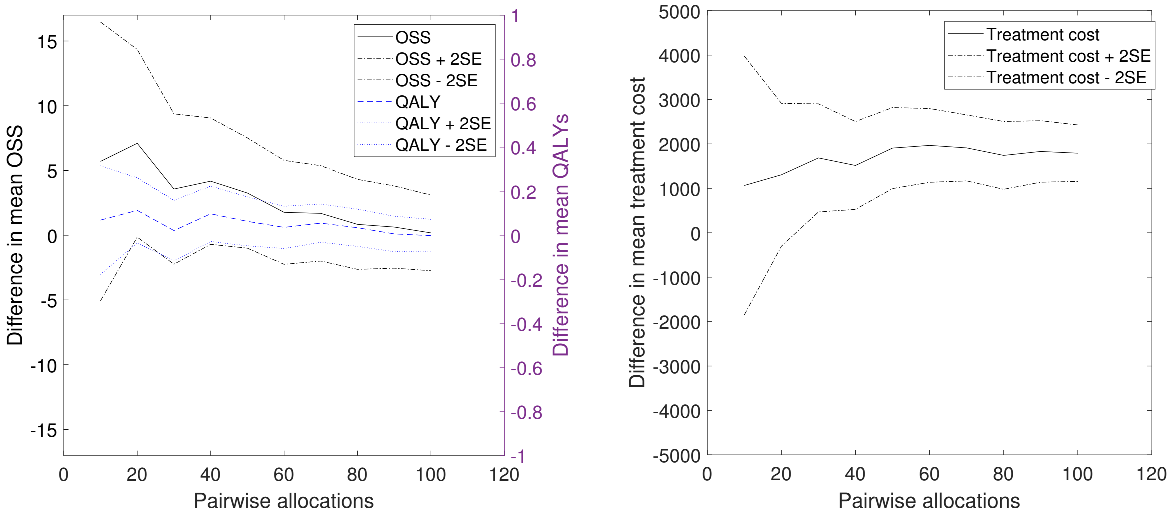

(a) Health outcomes (Oxford Shoulder Score and QALY). Values above zero show surgery to be superior to sling.

(b) Treatment cost. Values above zero show surgery to be more expensive than sling.

Figure SM.1: Differences between sample means for the health outcomes Oxford Shoulder Score and QALY and treatment cost, together with limits at $\pm$ two standard errors (SE).

Figures SM.1a and SM.1b show how the differences between the sample mean QALYs and treatment cost at one year – the two constituent parts of the average incremental net monetary benefit that is plotted in Figure 2 of the journal article – evolved as the sample size accumulated. The horizontal axis records the number of pairwise allocations, measured in blocks of ten pairwise allocations at a time, as in the journal article. Figure SM.1a shows that there was little difference between the sample mean QALYs as evidence accumulated: surgery was slightly superior to sling initially, but as the sample size accumulated the difference fell to zero. Also plotted in Figure SM.1a is the difference between the sample means of the primary health outcome measure, the Oxford Shoulder Score. Once more, there is some evidence favouring surgery early on, but it falls over time. A difference of five points in the Oxford Shoulder Score was deemed to be of clinical importance (Handoll et al., 2015). Figure SM.1a shows the maximum difference to be about 7, in favour of surgery, after 20 pairwise allocations.

Figure SM.1b shows the difference between the sample means for treatment cost. Surgery was estimated to be approximately £1,000 more expensive than sling initially, rising to just under £2,000 more expensive by the end of the study.

1.2.2 Assumptions regarding the choice of parameter values.

The parameter values used for the analysis are sourced and calculated as follows:

1. Estimate of the proportion treated with sling at the start of the trial, p_{N}: taken from Handoll et al. (2015, page 104). Of 313 non-consenters in the ProFHER trial, 66 were assigned to surgery, 105 to sling, 118 were classified as 'uncertain' and data were missing for the

remaining 24. Assume that non-consenters were representative of the overall patient population for which the ProFHER trial was designed and that patients with missing data for treatment, together with those classified as 'uncertain', do not systematically differ from the study population either. Then it is estimated that $p_{\mathrm{N}} = 0.39$ (= 66/171).

2. Estimate of switching costs: from personal communications it was believed that these costs would be minor and they are assumed to be equal to zero.

3. Estimate of the sampling variance, σ_X^2: from the 95% confidence interval for expected incremental net monetary benefit at one year provided by the ProFHER trial's data. The point estimate of expected incremental net monetary benefit at one year was -£1601.66 and the upper limit of the 95% confidence interval was -£458.06, based on approximately 60 pairwise allocations. Using a critical value of $t = 2$, $\sigma_X \approx \sqrt{60} \times$ (-£458.66 + £1601.66)$/2 \approx$ £4,400.

4. Estimate of P, the number of patients affected by the adoption decision: there appears to be no reliable information on the annual incidence rate of fractures meeting the inclusion criteria for the ProFHER trial. We therefore estimated P using information from a number of sources. Corbacho et al. (2016, page 7) report that there were 3,519 first listed consultant episodes for patients with fractures of the proximal humerus which involved an operation during 2011–12. They assume that 80% of these were displaced fractures involving the surgical neck. They make the conservative assumption that 50% of these cases may change from surgical intervention to non-surgical intervention as a result of the ProFHER trial and calculate a £2.5m saving to National Health Service England (i.e. 3,519 $\times$ 0.8 $\times$ 0.5 = 1,408 patients $\times \Delta C$ = £1,758 = £2.5m). Treatment using sling is classified as an outpatient appointment in the United Kingdom, and there are no data on the number of sling administrations that took place during 2011–12. Given that Corbacho et al. (2016) estimate that there were 2,815 (= 0.8 $\times$ 3,519) cases of fractures of the proximal humerus involving the surgical neck during 2011-12, we use p_{N} from point 1 above to estimate that 4,403 (2,815 $\times$ (1 / (0.39) - 1)) patients would have been treated with sling. We therefore estimate an annual incidence rate of 2,815 + 4,403 $\approx$ 7,000 patients who may be treated either with surgery or sling. We combine this with a total duration for implementing the decision resulting from the trial which is equal to 6 years, so P = 6 $\times$ 7,000 = 42,000.

5. Estimate of c, the marginal cost per pairwise allocation, is calculated using the financial records from the trial (those used to produce Figure 2 of the journal article). Approximately £161,000 was spent prior to recruiting the first patients. This is classified as the fixed set-up cost of the trial. An estimated 50% of the £1,020,000 of costs incurred between the start of patient recruitment and the finish of follow-up is taken to be the variable cost of the trial, giving an estimate of the marginal cost of adding one pairwise allocation to be £510,000/125 = £4,080. The remaining 50% is taken to be a cost (such as overheads) which would have been incurred during the recruitment phase even if no patients were being recruited. Finally, costs of £289,000 are incurred post follow-up. This gives a total spend of £1,470,000.

Table SM.2: Parameter values used for the model (pages SM.3 to SM.4 discuss the assumptions behind their chosen values).

Parameter	Definition	Value	Source
p_{N}	Proportion treated with surgery at the start of the trial	0.39	Handoll et al. (2015)
	Fracture incidence rate	7,000 patients per annum	Derived from Corbacho et al. (2016)
	Time horizon for post-decision population	6 years	Assumption
P	Population expected to benefit from adoption decision	42,000 patients	Defined from above parameters
σ_X	Standard deviation for incremental net monetary benefit in population	£4,400	ProFHER trial
n_0	Effective sample size of the prior distribution	2 pairwise allocations	Assumption
μ_0	Prior mean for expected incremental net monetary benefit	0	Assumption
Δ	Period over which quality of life data is followed up	1 year	Assumption
	Estimated annual rate of recruitment to trial	47 pairwise allocations	ProFHER trial
τ	Delay for observing health outcome if interest (in pairwise allocations)	47 pairwise allocations	Annual rate of recruitment
	Time horizon of trial	32 months	Handoll et al. (2015)
I_{N}	Cost of switching $P(1-p_{\mathrm{N}})$ patients to surgery	£0	Estimate
I_{S}	Cost of switching Pp_{N} patients to sling	£0	Estimate
	Estimated spend on fixed costs prior to starting trial	£161,000	ProFHER trial's accounts
	Estimated spend on fixed costs during trial	£510,000	ProFHER trial's accounts
	Estimated spend on variable costs	£510,000	ProFHER trial's accounts
	Estimated spend on fixed costs post follow-up	£289,000	ProFHER trial's accounts
c_{fixed}	Total spend on fixed costs	£960,000	ProFHER trial's accounts
	Total spend	£1,470,000	ProFHER trial's accounts
c	Estimated cost per pairwise allocation	£4,080	ProFHER trial's accounts
λ	Maximum willingness to pay for one QALY	£20,000	NICE (2013)

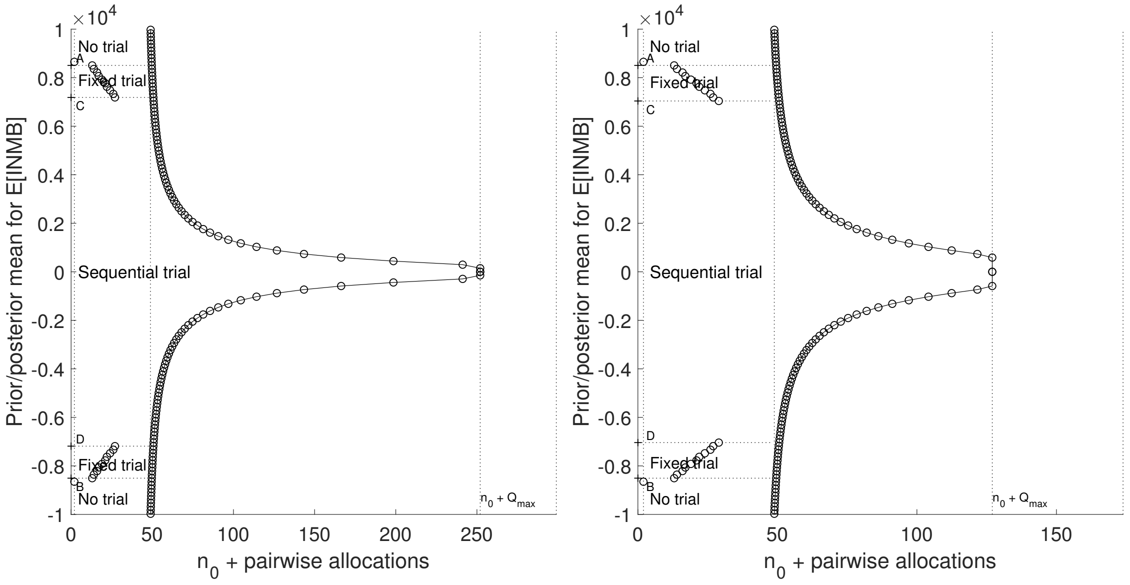

(a) $Q_{\text{max}} = 250$ pairwise allocations.

(b) Q_{max} =125 pairwise allocations.

Figure SM.2: Stopping boundaries showing the maximum length of Stage II, together with the optimal numer of pairwise allocations during Stage I, for $Q_{\text{max}} = 250$ and $Q_{\text{max}} = 125$ pairwise allocations.

2 Results

2.1 The stopping boundaries

Figure SM.2 shows the stopping boundaries for the two versions of the model in (n_0 + pairwise allocations × prior/posterior mean) space. The optimal Stage I decisions (run a sequential trial, run a trial with a fixed sample size, run no trial), delineated by the letters A–D as they were in Figure 1 of the journal article, are shown, together with circles showing some of the optimal pairwise allocations for the Optimal Bayes One Stage design.

2.2 Additional Monte Carlo simulation

We used additional Monte Carlo simulation to explore how the Optimal Bayes Sequential policy performs when the prior mean for the unknown expected incremental net monetary benefit is varied over a range of values, with sampling means drawn from the resulting prior distribution. We did this for three trial designs: the Optimal Bayes Sequential design, with $Q_{\text{max}} = 250$ and $Q_{\text{max}} = 125$, and a fixed sample size (i.e. non-sequential) version with 125 pairwise allocations, which we call the 'Fixed' design.

We used a range of values of the prior mean, between a lower limit of −£10,000 and an upper limit of £10,000. For each value, we took the value of n_0 used for the bootstrap analysis and made 15,000 random draws of W from the resulting distribution. For each draw, we sampled

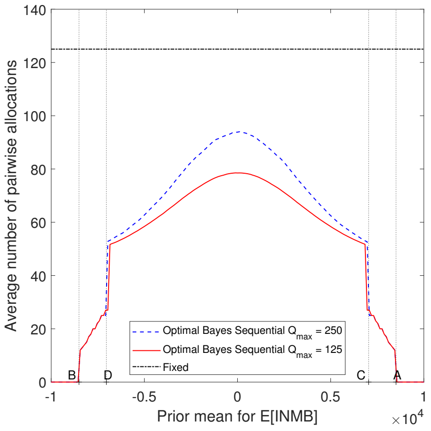

(a) Average number of pairwise allocations.

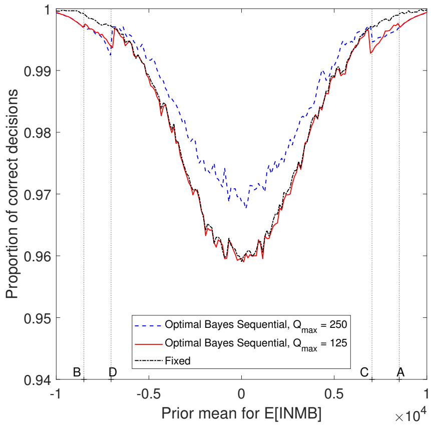

(b) Proportion of correct decisions.

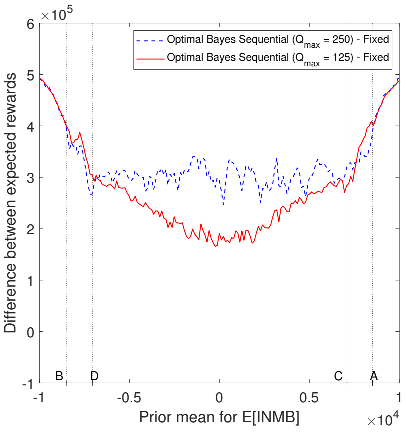

(c) Estimate of difference between expected benefits.

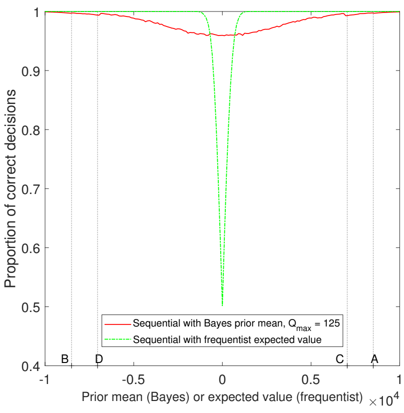

(d) Proportion of correct decisions: comparison of Optimal Bayes Sequential with sequential approach when $\sigma_0 = 0$.

Figure SM.3: Results from the Monte Carlo simulation.

at random from the sampling distribution and used these draws to generate a path for the posterior mean. For each path we calculated the stopping time of the trial and the adoption decision made at the end of Stage III. We investigated performance characteristics including the average number of pairwise allocations, the probability of making the correct selection decision[1] and the average benefit. Results are presented in Figures SM.3a to SM.3d.

Figure SM.3a presents the average sample size of the Optimal Bayes Sequential design as a function of the prior mean for the expected incremental net monetary benefit. The letters 'A'–'D' correspond to those marked in Figure 1 of the journal article. Figure SM.3a shows that, for both sequential designs, the average sample size is lower than the 125 pairwise allocations used for the Fixed design and it falls the further the prior mean is from zero, which is to be expected. Comparison of the Optimal Bayes Sequential design when $Q_{\max} = 125$ with that when $Q_{\max} = 250$ shows that doubling the maximum sample size increases the average sample size

most at $\mu_0 = 0$ (from about 79 pairwise allocations to about 94). So, compared with running a fixed sample size trial with 125 pairwise allocations, the two Optimal Bayes Sequential designs are expected to reduce the sample size of the trial.

Figure SM.3b presents the proportion of times that each design makes the correct technology selection decision. The Fixed design and the Optimal Bayes Sequential design with $Q_{\text{max}} = 125$ pairwise allocations have a very similar performance. Doubling the maximum sample size of the Optimal Bayes Sequential design increases the proportion of correct decisions by about one percentage point, reflecting the value of continuing to learn about W. Performance of all three designs is worst in the region of $\mu_0 = 0$, although the correct selection is still made in approximately 96–97% of the replications. So, although the two Optimal Bayes Sequential designs are expected to reduce the sample size (Figure SM.3a), there is little impact on the probability that the correct technology is selected.

Figure SM.3c plots the estimate of the 'net gain' of the Optimal Bayes Sequential designs over the Fixed design. Net gain is defined as the difference between the average benefit of the Optimal Bayes Sequential design and the Fixed design, accounting for both the benefit accruing at the point of technology selection and the cost of the trial. The two sequential designs outperform the Fixed design by between just under £200,000 and just over £300,000, owing to the fact that they both save costs through early stopping, on average (Figure SM.3a), while making a similar proportion of correct decisions (Figure SM.3b).

Finally, Figure SM.3d compares the proportion of correct decisions made by the Optimal Bayes Sequential design when $Q_{\text{max}} = 125$ pairwise allocations (the same red, continuous line that is plotted in Figure SM.3b) with the proportion of correct decisions from what we term a 'frequentist' approach to the Monte Carlo simulation (green, dash-dot line). For the frequentist approach, σ_0 is set equal to zero, so that the sampling mean is no longer a random draw from a prior distribution but is equal to the prior mean for all 15,000 replications. Figure SM.3d shows that, when $\mu_0 = W = 0$, the probability of selecting surgery is equal to one half owing to the fact that the stopping boundary is symmetric, but it increases the further W lies from 0.

2.3 Additional sensitivity analysis

Additional sensitivity analysis may be used to investigate the impact of changes in parameter values on the stopping boundary, the ranges of μ_0 over which the 'no trial', Optimal Bayes One Stage and the Optimal Bayes Sequential designs are optimal, as well as the operating characteristics. Holding a given set of parameter values constant, Chick et al. (2017b, Sections S.6.1 and S.6.2) show that reducing the delay, τ, reduces the range over which running the Optimal Bayes One Stage design is optimal. Increasing the variable research cost, c, shrinks the Stage II continuation region and reduces the range of the prior mean over which the Optimal Bayes Sequential design is optimal. Increasing P has the opposite effect: a higher value of P widens the continuation region and makes the sequential trial more attractive. Further, it may be shown that increasing c_{fixed} moves point 'A' towards 'C' and point 'B' towards 'D' in Figure 1 of the journal article, increasing the region over which 'no trial' is optimal, because running no trial incurs no fixed cost. If fixed costs are high enough, the region over which the Optimal Bayes Sequential design is optimal shrinks (points 'C' and 'D' move towards 0). Comparison of value

functions in this manner, accounting for the fixed costs of operating the trial, therefore offers the potential for informing design decisions at the research commissioning stage.

Notes

[1]A ‘correct selection decision’ is defined according to the value of the draw for W. An adoption decision is defined as being correct if $W > 0$ and the posterior mean informing the adoption decision suggests surgery is cost-effective or if $W \leq 0$ and the posterior mean informing the adoption decision suggests sling is cost-effective.

References

Alban, A., Chick, S. E., and Forster, M. (2020). Value-based clinical trials: selecting trial lengths and recruitment rates in different regulatory contexts. Discussion Papers 20/01, Department of Economics, University of York.

Chick, S. E., Forster, M., and Pertile, P. (2017a). A Bayesian decision-theoretic model of sequential experimentation with delayed response. *Journal of the Royal Statistical Society, Series B*, 79(5):1439–1462.

Chick, S. E., Forster, M., and Pertile, P. (2017b). Online supplementary material for Chick et al. (2017).

Corbacho, B., Duarte, A., Keding, A., et al. (2016). Cost effectiveness of surgical versus non-surgical treatment of adults with displaced fractures of the proximal humerus. *Bone and Joint Journal*, 92-B(2):152–159.

Handoll, H., Brealey, S., Rangan, A., et al. (2015). The ProFHER (PROximal Fracture of the Humerus: Evaluation by Randomisation) trial - a pragmatic multicentre randomised controlled trial evaluating the clinical effectiveness and cost-effectiveness of surgical compared with non-surgical treatment for proximal fracture of the humerus in adults. *Health Technology Assessment*, 19:1–280.

NICE (2013). Guide to the methods of technology appraisal. https://www.nice.org.uk/process/pmg9/chapter/foreword (Accessed 19th January, 2021).
